# Genome-wide sequencing and metabolic annotation of *Pythium irregulare* CBS 494.86: understanding Eicosapentaenoic acid production

**DOI:** 10.1186/s12896-019-0529-3

**Published:** 2019-06-28

**Authors:** Bruna S. Fernandes, Oscar Dias, Gisela Costa, Antonio A. Kaupert Neto, Tiago F. C. Resende, Juliana V. C. Oliveira, Diego M. Riaño-Pachón, Marcelo Zaiat, José G. C. Pradella, Isabel Rocha

**Affiliations:** 1 Department of Civil and Environmental Engineering, Federal University of Pernambuco, Recife, PE Brazil; 2 Centre of Biological Engineering, Universidade do Minho, Braga, Portugal; 3 Brazilian Bioethanol Science and Technology Laboratory (CTBE), Brazilian Centre of Research in Energy and Materials (CNPEM), Campinas, SP Brazil; 4 Computational, Evolutionary and Systems Biology Laboratory, Center for Nuclear Energy in Agriculture, University of São Paulo, Piracicaba, São Paulo, Brazil; 5 Biological Processes Laboratory, Center for Research, Development and Innovation in Environmental Engineering, São Carlos School of Engineering (EESC), University of São Paulo, São Carlos, SP Brazil; 6 PRBiotec Ltd, São José dos Campos, SP Brazil

**Keywords:** Eicosapentaenoic acid, Metabolic annotation, *Pythium irregulare*, unsaturated fatty acids, whole-genome sequence

## Abstract

**Background:**

*Pythium irregulare* is an oleaginous Oomycete able to accumulate large amounts of lipids, including Eicosapentaenoic acid (EPA). EPA is an important and expensive dietary supplement with a promising and very competitive market, which is dependent on fish-oil extraction. This has prompted several research groups to study biotechnological routes to obtain specific fatty acids rather than a mixture of various lipids. Moreover, microorganisms can use low cost carbon sources for lipid production, thus reducing production costs. Previous studies have highlighted the production of EPA by *P. irregulare*, exploiting diverse low cost carbon sources that are produced in large amounts, such as vinasse, glycerol, and food wastewater. However, there is still a lack of knowledge about its biosynthetic pathways, because no functional annotation of any *Pythium* sp. exists yet. The goal of this work was to identify key genes and pathways related to EPA biosynthesis, in *P. irregulare* CBS 494.86, by sequencing and performing an unprecedented annotation of its genome, considering the possibility of using wastewater as a carbon source.

**Results:**

Genome sequencing provided 17,727 candidate genes, with 3809 of them associated with enzyme code and 945 with membrane transporter proteins. The functional annotation was compared with curated information of oleaginous organisms, understanding amino acids and fatty acids production, and consumption of carbon and nitrogen sources, present in the wastewater. The main features include the presence of genes related to the consumption of several sugars and candidate genes of unsaturated fatty acids production.

**Conclusions:**

The whole metabolic genome presented, which is an unprecedented reconstruction of *P. irregulare* CBS 494.86, shows its potential to produce value-added products, in special EPA, for food and pharmaceutical industries, moreover it infers metabolic capabilities of the microorganism by incorporating information obtained from literature and genomic data, supplying information of great importance to future work.

**Electronic supplementary material:**

The online version of this article (10.1186/s12896-019-0529-3) contains supplementary material, which is available to authorized users.

## Background

*Pythium irregulare* is an oleaginous diploid Oomycete, a microscopic Stramenopiles [1, 2] and pathogen of various crops [1], including *Arabidopsis* plants [3]. *P. irregulare* has the potential to be industrially used to produce lipids because it is able to accumulate a large amount of these compounds, including Eicosapentaenoic acid (EPA) [4]. EPA (C_20_H_30_O_2_) is a 20-carbon polyunsaturated fatty acid with five *cis* double bonds, with the first double bond located at the third carbon from the omega end, which justifies its classification as an omega-3 fatty acid. The Food and Agriculture Organization of the United Nations recommends ingestion up to 500 mg per day of EPA and DHA (Docosahexaenoic acid) in the early years of life and for prevention of cardiovascular diseases [5], as it is not naturally synthesized in humans. Omega-3 fatty acids are important dietary supplements, with high selling prices (US$ 600 – US$ 4000 per kg of omega-3) [6], and a promising and very competitive market [7]. The expected omega-3 revenue is estimated at US$ 2.7 billion by 2020, with a Compound Annual Growth Rate (CAGR) of 17.5% (2014–2020), just in the pharmaceutical market [8]. This scenario has prompted several groups to search for alternative ways to produce omega-3, particularly EPA. Microorganisms are very attractive sources of EPA, because they can be driven to produce specific fatty acids rather than a mixture of various lipids, using low cost carbon sources without presence of heavy metals in the cultivated medium. This can reduce the cost of lipid extraction and purification and help to reduce the dependence on fish-oil. Some microorganisms have been studied with this goal, such as *Mortierella alpine*, *Mortierella elongate*, *Monochrysis luteri, Pseudopedinella sp.*, *Coccolithus huxleyi*, *Cricosphaera carterae*, *Monodus sub-terraneous*, *Nannochlorus sp.*, *Porphyrium cruentum*, *Cryptomonas muculata*, *Cryptomonas sp.*, *Rhodomonas leans,* and *Pythium irregulare* [9–12].

Some studies have indicated the possibility of producing EPA using *P. irregulare*, exploiting diverse abundant low-cost carbon sources including wastewaters such as vinasse from corn-meal ethanol production, glycerol, wastewater from the food industry, and several sugars [4, 13, 14]. However, there is still a lack of knowledge about the biosynthetic pathways for EPA in this microorganism, and Stramenopiles, in general. This taxonomical order covers very diverse ecological niches and lifestyles ranging from photosynthetic diatoms and brown algae to filamentous saprophytic and pathogenic oomycetes [15].

Hereto, *Pythium irregulare* DAOM BR486 is the only *P. irregulare* strain sequenced and annotated at the National Center for Biotechnology Information - NCBI database (Bioproject number: PRJNA169053). Its annotation was performed automatically using MAKER v.203 tool [16] and was based on *Pythium ultimum* Genome database [17] [18]. However, it aimed at evaluating the pathogenicity of oomycetes, disregarding the annotation of metabolic functions. Moreover the automatic annotation can produce false positive and erroneous data [19].

The goal of this work was to identify the key genes and pathways related to EPA biosynthesis, as well as other metabolites of biotechnological importance, in *P. irregulare* strain CBS 494.86, including amino acids and fatty acids production, and consumption of carbon and nitrogen sources, present in the wastewater. As this strain was unexplored and unpublished, its genome was thus sequenced and annotated, with a special emphasis in metabolic functions that were thoroughly manually curated. Its possible application was examined through a biotechnological perspective, using as carbon sources vinasse from bioethanol production process a low cost wastewater produced globally in high amount, such as vinasse, from bioethanol process production, glycerol, from biodiesel wastewater (obtained in the biodiesel production), and several food and beverage wastewaters (Additional file 1: Figure S1).

The genome-wide functional annotation, presented in this manuscript, was corroborated with evidences from literature, thus allowing its use as the basis for the reconstruction of a genome scale metabolic model.

## Results

### Whole-genome sequencing

The species classification of the isolate selected for genome sequencing was confirmed by Sanger sequencing and analysis of the cytochrome oxidase I gene (COI) and internal transcribed spacer regions (ITS1 and ITS2), which were aligned, using the nucleotide Basic Local Alignment Search Tool (BLAST) [20], with the NCBI genomic database. The ITS sequence was 98% identical to *P. irregulare* CBS 250.28 (sequence ID: AY598702.2) with coverage of 86%, wheras the COI sequence was 99% identical to *P. irregulare* CBS 493.86 / CBS 250.28 (sequence ID: GU071821.1) with coverage of 99%.

The genomic DNA from the cultivated *P. irregulare* strain CBS 494.86 was extracted and sequenced on a HiSeq2500 using a single paired-end library (2x100bp). The HiSeq2500 produced 58,990,406 sequenced fragments 2x100bp which were used for assembly; 43,436,209 of these remained after quality control. Genome assembly resulted in 9658 scaffolds larger than 500 bp, with an N50 of 13.460 bp (6.653 scaffolds longer than 1 Kbp) and a total genome size of 47.121.789 bp (Table 1). The coverage evaluation of the gene space by our assembly was performed using BUSCÒ v3 [21] with two sets of conserved genes, one for all eukaryotes with 303 conserved genes and one for protists with 215 conserved genes. For both datasets our assembly showed over 90% of coverage of complete BUSCÒs (Additional file 2). The gene space coverage observed in this project assembly is similar to that of the published genome sequence of *P. irregulare* strain DAOM BR486, and the size of their haploid genomes is also similar [21] (Additional file 2). Gene prediction was carried out with Augustus [22], which was trained by exploiting available data from the Buell lab of another strains of *P. irregulare and P. ultimum* [23] [17], and resulted in the prediction of 17,008 protein-coding genes and 29 tRNA genes. The different copies of the ribosomal operon were collapsed into a single copy. In strain CBS 493.86, 95.4% of the predicted genes can be mapped to the genome of strain CBS 805.95. The sequenced genome was deposited in NCBI (Bioproject number: PRJNA371716).

**Table 1:** *Pythium irregulare* CBS 494.86 genome statistics

Assembly statistics for genome	
Estimated genome size	47.1 Mb
Number of scaffolds	9658
Number of scaffolds (≥ 1000 bp)	6653
Number of scaffolds (≥ 5000 bp)	2204
Number of scaffolds (> = 10,000 bp)	1192
Number of scaffolds (≥ 25,000 bp)	334
Number of scaffolds (≥ 50,000 bp)	64
Total length	45,784,433
Total length (≥1000 bp)	43,603,753
Total length (≥ 5000 bp)	33,709,764
Total length (≥ 10,000 bp)	26,550,523
Total length (≥ 25,000 bp)	13,047,962
Total length (≥ 50,000 bp)	4,077,371
Largest scaffolds	191,247
GC (%)	53.43
Scaffolds N50	13,460
Scaffolds N75	4686
Scaffolds L50	878
Scaffolds L75	2334
# N’s per 100 kbp	262.38
Number of genes	17,758
Number of mRNAs	17,727
Number of tRNAs	29
Number of rRNAs	2
Total CDS length	23,740,968
Total Gene length	23,750,652
Average gene length	1337.54
Longest gene	26,310
Shortest gene	42
% of genome covered by genes	51.87
% of genome covered by CDS	51.85
Average number of exons	2.58
Max number of exons	42

### Metabolic annotation

The *merlin* software (metabolic models reconstruction using genome-scale information) [24] was used for the functional annotation of proteins with metabolic functions encoded in the genome of *P. irregulare*.

For the analysis and interpretation of the results from the semi-automatic annotation performed by *merlin*, each candidate metabolic gene was inspected and accepted, or rejected, according to a developed annotation pipeline, reported in the Methods section.

The manual curation of *merlin* results began by inspecting the information in different databases for each candidate and identified homologues, prioritizing UniProt’s reviewed information [25]. As the functional annotation of *P. irregulare* is yet to be described and most annotations in the Stramenopiles lineage are not reviewed in UniProt, the annotation pipeline took into account phylogeny [26], to retrieve the closest organisms with reviewed information at Swiss-Prot.

*Arabidopsis thaliana* was defined as the organism of reference in the annotation process, since Stramenopiles are the closest relatives of Viridiplantae [27]. Moreover *Arabidopsis thaliana* has been widely used in genomic studies, for example by Arabidopsis Genome Initiative, AGI, since 1996 [28], affording high consistency in the *P. irregulare* annotation.

From the 17,727 candidate genes provided by the genome sequencing, 5213 were found to have homologies with metabolic genes. From those, 2622 candidates (50.3%) had very high confidence level, meaning that these genes have a very high probability of being correctly classified, because there was consistency in the Enzyme Commission (EC) numbers found in the similarity search conducted. On the other hand, there were 1404 gene candidates with very low confidence level, which means that these might have been erroneously assigned with metabolic functions, weakening the classification confidence, and consequently they were rejected from the set of metabolic genes. There were also 1187 candidates with high, medium and low confidence level, which were manually curated, according to the developed pipeline described in the Methods section (Fig. 8).

**Fig. 1:**
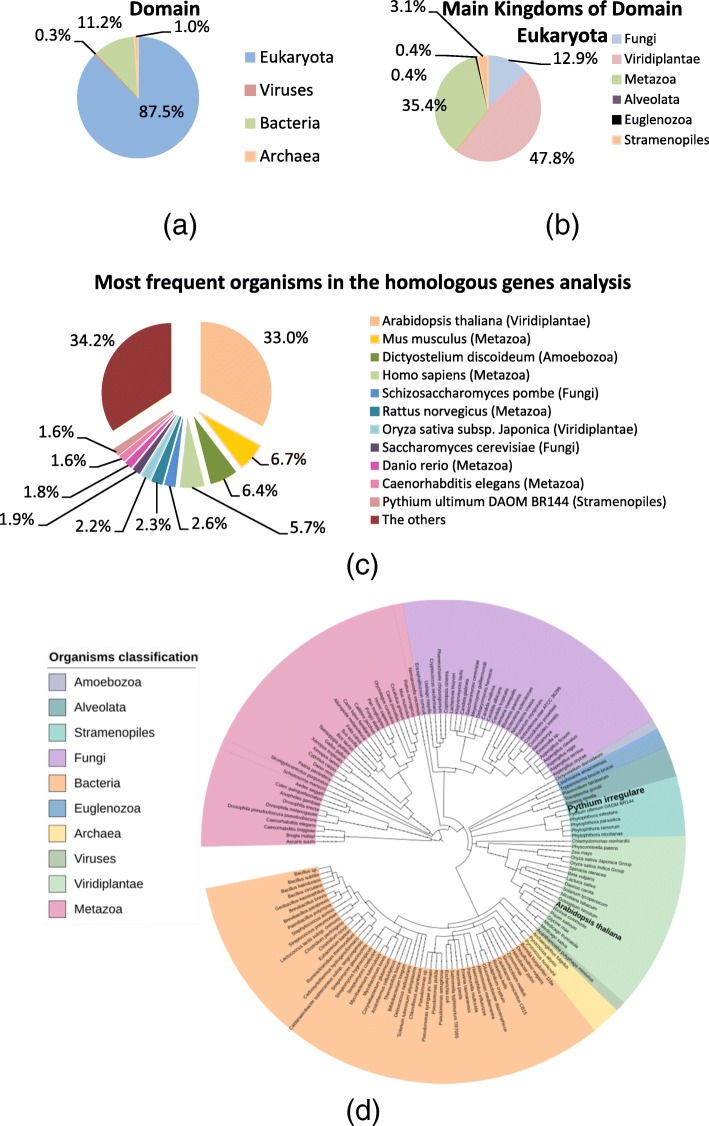
Analysis of 470 organisms reported in the set of homologues of the candidate genes. Detailed legend: Analysis developed with merlin for the enzymatic annotation (**a**) Distribution by domains; (**b**) Distribution by the main Kingdoms of Domain Eukaryota; (**c**) The most frequent organisms and their Kingdoms in the analysis of homologous of candidate gene; and (**d**) Phylogenetic Tree of the organisms reported in the set of homologous of the candidate genes (reported at least twice – 154 organisms). The figure was drawn using the iTOL tool [29]

Figure 1 displays the distribution of organisms with at least one homologous gene found during the enzymatic annotation, and its domain or kingdom of origin. A total of 9338 organisms were mapped in the homologous gene analysis, with 470 of them being reported in the enzymatic annotation, 154 of which were reported at least twice in the annotation. Among the 470 organisms, 87.5% were from Eukaryota Domain (Fig. 1.a), predominating the Viridiplantae Kingdom (Fig. 1.b), with 34.3% of homologue genes coming from *Arabidopsis thaliana*. Other organisms listed do not exceed 7% of frequency; for example, *P. ultimum*, a Stramenopiles microorganism, was only listed in 1.6% of the cases (Fig. 1.c). These results corroborated the choice of *Arabidopsis thaliana* in the pipeline development. Moreover, the Phylogenetic tree represented in Fig. 1.d shows high taxonomic similarity between Viridiplantae and Stramenopiles, according to NCBI taxonomy identifiers.

**Fig. 2:**
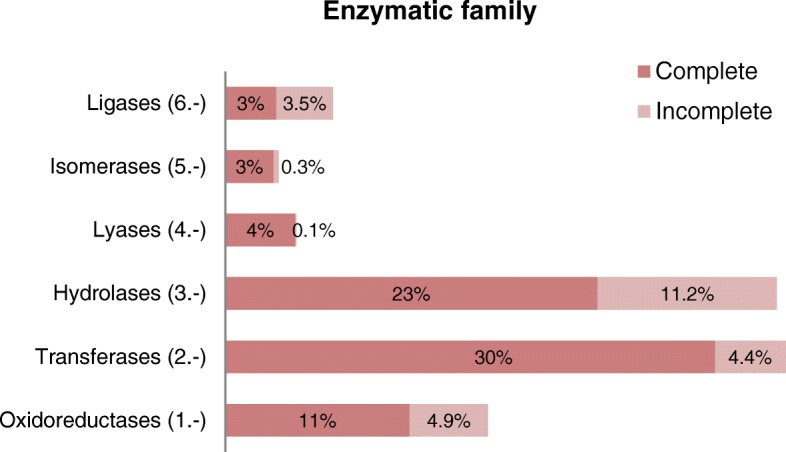
Classification of genes according to the enzymatic family obtained in the *P. irregulare* metabolic annotation

According to the developed pipeline, 3852 EC numbers were manual and automatic assigned (Fig. 2). The enzyme class distribution is described in Fig. 2. Transferases and hydrolases are the biggest enzyme groups with 35 and 34% of annotated EC numbers, respectively. On the other hand, lyases, isomerases, and ligases, only encompass 4, 3, and 7% of the annotated EC numbers, respectively. Figure 2 shows that 24.4% of the annotated EC numbers are partial, with hydrolases as the group having the largest amount of incomplete EC numbers (11.2%).

Regarding the annotation of transporters, *merlin’s* TRIAGE [30] independent module identified candidate transporter proteins encoding genes and, for the genes that fulfilled certain conditions, automatically created transport reactions. From this annotation, 945 candidate genes were identified to encode membrane transporter proteins associated with the transport of 860 metabolites. These data are available in the Availability of data and materials (Additional file 4). TRIAGE classified 39.9% genes as Electrochemical Potential-driven Transporters (transporter classification (TC) TC2), 24.4% as Channel/Pores (TC1), 20.2% as Primary Active Transporters (TC3), 9% as Incomplete Characterized Transport Systems (TC9), 4% as Accessory Factors Involved in Transport (TC8), 1% as Group Translocators (TC4), and 1% Transmembrane Electron Carriers (TC15) (Table 2).

**Table 2:** Transporters level and classification (TC) associated with candidate genes identified by *merlin*’s TRIAGE

TC level	Classification	Number of TCG^a^	Percentage (%)
TC1	Channels/Pores	231	24.4
TC2	Electrochemical Potential-driven Transporters	377	39.9
TC3	Primary Active Transporters	191	20.2
TC4	Group Translocators	8	0.9
TC5	Transmembrane Electron Carriers	13	1.4
TC8	Accessory Factors Involved in Transport	36	3.8
TC9	Incompletely characterized Transport Systems	89	9.4
Total		945	100

^a^*TCG* Transporter Candidate Genes

### Analysis of the functional annotation

#### Carbon source metabolism

The functional annotation of *P. irregulare* CBS 494.86 was compared with curated information for *Arabidopsis thaliana*, *Saccharomyces cerevisiae*, *Yarrowia lipolytica*, and *Mortierella alpina* [28, 31–37], because there is no curated metabolic annotation available for any *Pythium* strain. The reason for choosing *A. thaliana* was already mentioned above, while *S. cerevisiae*, *Y. lipolytica*, and *M. alpina* are relevant producers of lipids of commercial interest, are well characterized in the literature and are considered promising EPA producers [9]. Additionally, Oomycetes are generally not employed for lipids production, except *P. irregulare*. As this is the first curated functional annotation developed for *Pythium irregulare*, all metabolic pathways presented in the present article are mostly based on the findings obtained through the metabolic annotation and crossed with evidence from the literature.

In general, there were high similarity between glycolysis, pentose phosphate, and tricarboxylic acid (TCA) cycle pathways for *P. irregulare*, *A. thaliana*, *S. cerevisiae*, *Y. lipolytica,* and *M. alpina*. None of these organisms are able to perform the Entner-Doudoroff pathway, although this has been described for some Stramenopiles microorganism [38].

**Fig. 3:**
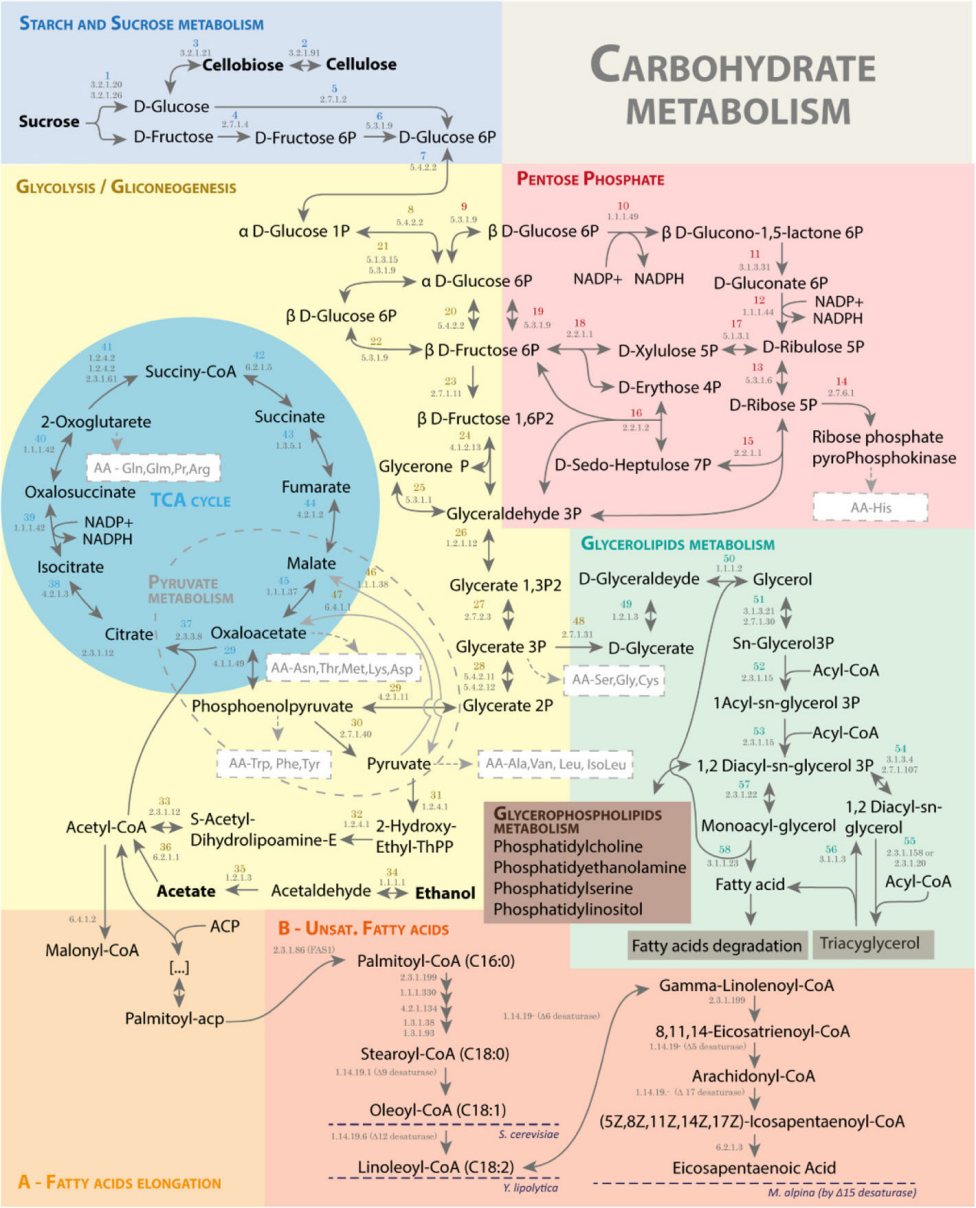
Carbon source consumption and Eicosapentaenoic acid biosynthesis simplified pathway based on the metabolic annotation. Detailed legend: The figure represents metabolites, reactions and enzyme or transporter annotated for each reaction. The figure is divided by pathways, and the pathways marked with A (Fatty acids biosynthesis) and B (Unsaturated Fatty acids biosynthesis) are described in Figura 7. The dashed blue lines (pathway B) highlights the end of fatty acids production by *Saccharomyces cerevisiae*, *Yarrowia lipolytica*, and *Mortierella alpina*

According to the illustration of the metabolic annotation shown in Fig. 3, the process of fatty acids biosynthesis starts with transport of some carbon source, such as sucrose, glucose, fructose, cellulose or glycerol. In the sucrose metabolism, for example, sucrose is degraded extracellularly by an irreversible reaction into D-fructose and D-glucose (by maltase-glucoamylase; EC:3.2.1.20 or beta-fructofuranosidase; EC:3.2.1.26) which will then be transported into the cell. Cellulose, as carbon source, is metabolized in cellobiose (by cellulose 1,4-beta-cellobiosidase; EC:3.2.1.91) and converted into D-glucose (by beta-glucosidase; EC:3.2.1.21), which is then phosphorylated to D-glucose-6P (by glucokinase; EC:2.7.1.2), entering into the Glycolysis pathway. Cellulose and cellobiose are not consumed by *Saccharomyces cerevisiae* or *Yarrowia lipolytica*. Glycerol is an example of a carbon source that comes from the glycolipid metabolism pathway (by glycerol kinase, EC: 2.7.1.30), which is converted by some reactions into 3-Phospho-D-glycerate to enter Glycolysis.

*P. irregulare* is a microorganism that could transport and use carbon sources available in different waste streams, namely, vinasse, from first or second generation ethanol production, composed by sucrose, D-glucose, D-fructose, glycerol, acetate, cellulose and others (Additional file 1: Figure S1) [4, 39–41]; glycerol, a by-product in biodiesel production process; and wastewaters from several food and beverage industries [42–44]. The results regarding the enzymes and transporters annotations are in agreement with the carbon sources reported in the literature [4, 13, 14, 29, 38] and presented in Table 3, with the exception of D-Galactose, L-rhamnose, and D-mannose. Though there is experimental evidence [14] regarding the use of these carbon sources by *P. irregulare*, and transporter proteins were identified for these sugars, no further consuming enzymes have been found in the metabolic annotation (Table 3).

**Table 3:**
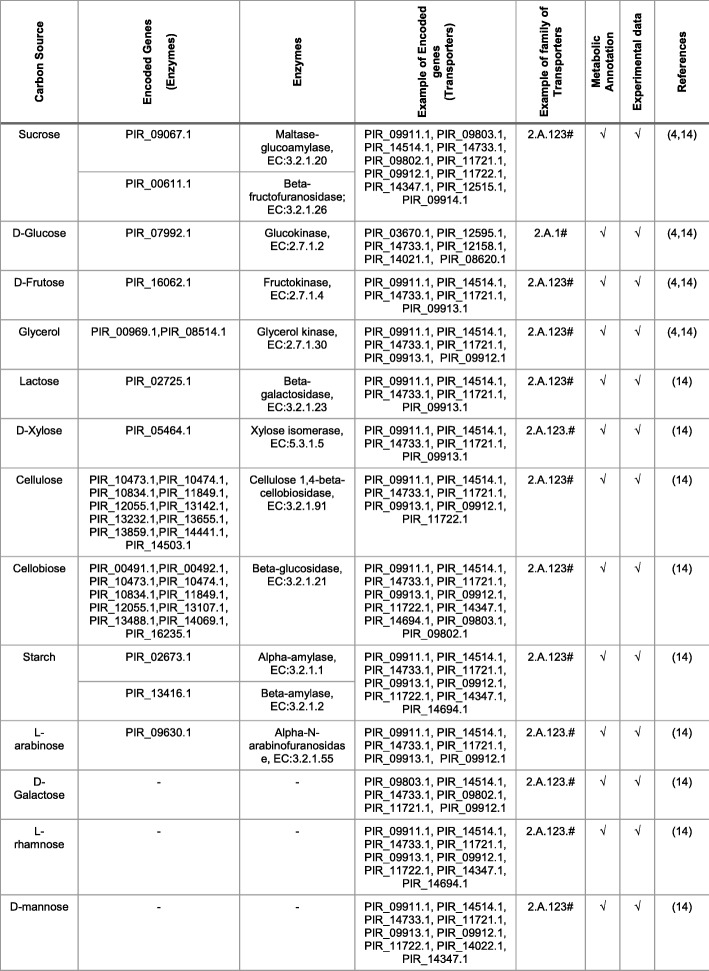
Comparison betwwen carbon source consumption based on the Metabolic Annotation and experimental data

Cellulose consumption is associated with the pathogenicity of *P. irregulare*, which is able to degrade plant cell walls of a wide range of plants (corn, soybeans, wheat, fruit trees, vegetables, cereals, and others). *P. irregulare* invades and forms haustoria within living plant cells, consuming its nutrients [23, 29, 45, 46]. The mapped candidate genes for cellulose consumption are important to understand the genetic basis of its pathogenicity.

In summary, most carbon sources (Fig. 3) are converted and guided to the Glycolysis pathway providing pyruvate, and subsequently acetyl-CoA, which, together with NADPH produced in the pentose phosphate pathway are critical precursors of fatty acid synthesis. Furthermore, other pathways can participate in acetyl-CoA’s availability, mainly consumption of amino acids from the host. Those pathways, together with the biosynthetic pathways for amino acids are reported in the following subsections.

#### Amino acids

This subsection aims to present the metabolic annotation obtained for amino acid production and highlight the main pathways involved in the fatty acid production.

##### General view

**Fig. 4:**
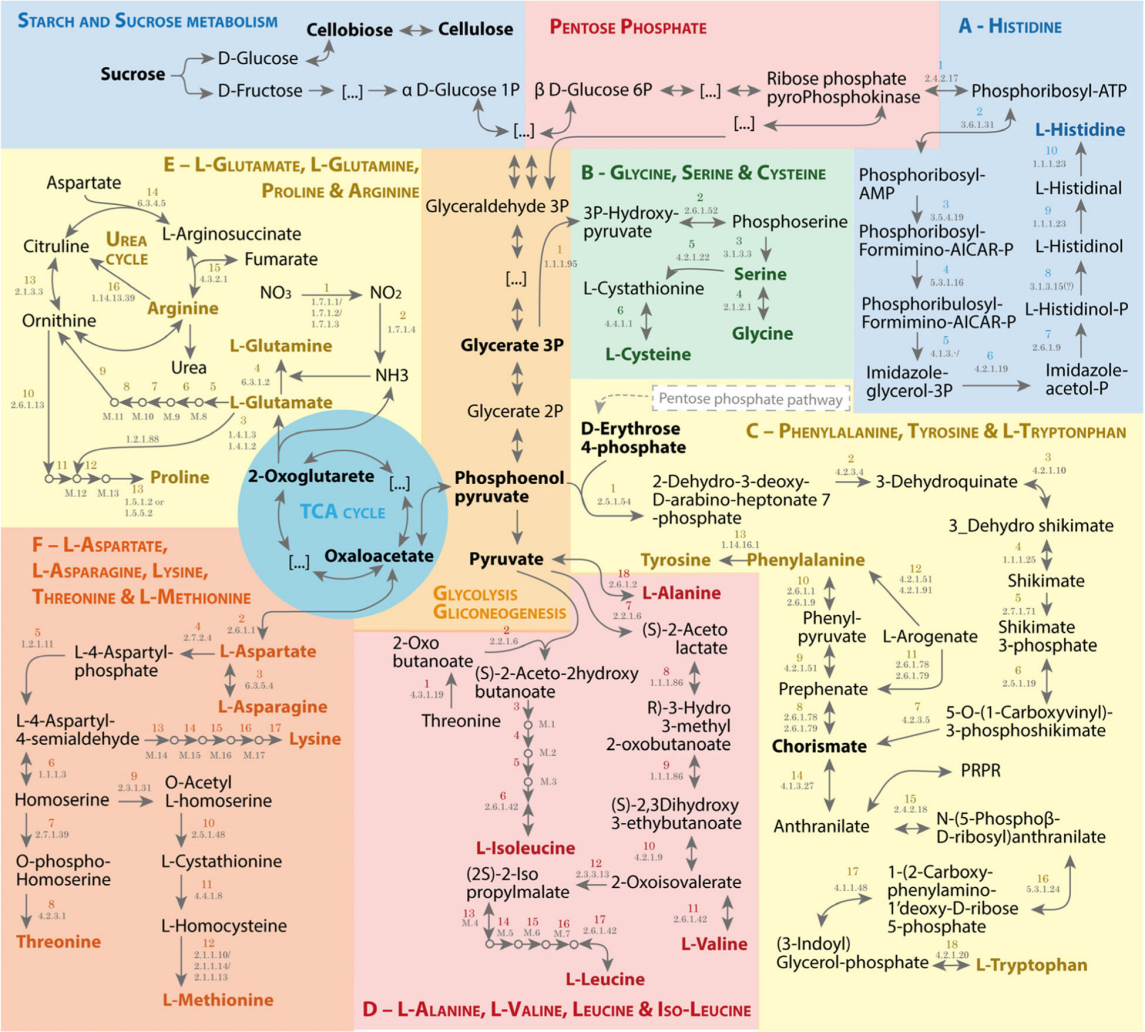
Prediction of amino acid biosynthesis according to the metabolic annotation of *P. irregulare* CBS 494.86. The amino acid pathways are listed from A to F. The reactions are enumerated inside each specific pathway, followed by the encoded EC number. The EC numbers with a question mark (?) are cases of reactions not predicted by the metabolic annotation. Some metabolites and reactions are marked with M (metabolites) and/or numbers decoded as follows: in D- L-Alanine, L-Valine, Leucine & Iso-Leucine pathway, M.1 = (R)-3-Hydro 3-methyl 2-oxopentanoate, M.2 = (S)-2,3 Dihydroxy 3-methypentanoate, M.3 = (S)-3-Methyl 2-oxopentanoate, M.4 = 2-Isopropylmaleate, M.5 = (2R,3S)-3-Isopropylmalate, M.6 = (2S)-2-Isopropyl 3-oxopentanoate, M.7 = 4-Methyl 2-oxopentanoate; 3 = EC:1.1.1.86; 4 = EC: 1.1.1.86; 5 = EC: 4.2.1.9; 13 = EC: 2.3.3.13, 14 = EC: 4.2.1.33; 15 = EC: 1.1.1.85; 16 = Spontaneous; in E – L-Glutamate, L-Glutamine, Proline & Arginine pathway, M.8 = N-Acetyl-glutamate, M.9 = N-Acetyl-glutamyl-P, M.10 = N-Acetylglutamate semialdehyde, M.11 = N-Acetyl-ornithine, 5 = EC: 2.3.1.1; 6 = EC: 2.7.2.8; 7 = EC: 1.2.1.38; 8 = EC: 2.6.1.11; 9 = EC: 3.5.1.14; in E – L-Glutamine, L-Glutamate, Proline & Arginine pathway, M.12 = L-Glutamate 5-semialdehyde; M13 = (S)-1-Pyrroline-5-carboxylate;11 = non-enzymatic; 12 = EC:1.5.1.2; in F – L-Aspartate, L-Asparagine, Lysine, Threonine & L-Methionine pathway, M.14 = (2S,4S)-4-hydroxy 2,3,4,5 tetrahydro-dipicolinate, M.15 = L-2,3,4,5- Tetrahydro-dipicolinate, M.16 = L-L-2,6 Diamino-pimelate, M.17 = meso2,6 Diamino-pimelate, 13 = EC: 4.3.3.7;14 = EC: 1.17.1.8; 15 = EC: 2.6.1.83 (?); 16 = EC: 5.1.1.7 (?); 17 = EC: 4.1.1.20

Figure 4 represents the possible pathways of amino acid production in *P. irregulare* according to its functional annotation developed in this research. In general, there are only a few differences in the amino acid production pathways reported for *A. thaliana* [28], some diatoms (*Thalassiosira pseudonana* and *Phaeodactylum tricornutum*), Stramenopiles microorganisms [47], and *S. cerevisiae* [35]. The divergences are in the pathways B – Glycine and Serine, C - Tyrosine, and E – Arginine, reported in this item (Fig. 4); and B – Cysteine and F –Lysine pathways, both described in item 2.3.2.1.1. Finally, a reflection regarding the pathways associated with fatty acids metabolism such as Leucine, Isoleucine, and Lysine degradation and Cysteine and Lysine biosynthesis is described in item 2.3.2.2 **Amino acids associated with metabolism of fatty acids (FAs) .**

##### B – Glycine and Serine pathways

*A. thaliana* and Diatoms have glycine and serine synthesis as part of photorespiration and non-photorespiration [47]. Only the non-photorespiratory pathway of serine synthesis is observed in *P. irregulare,* based on the functional annotation, similarly to what is observed in *S. cerevisiae* [35] and *Y. lipolytica* [34]*,* as well as other Stramenopiles [47] (Fig. 4).

##### C - Tyrosine pathway

Phenylalanine, tyrosine, and tryptophan are aromatic amino acids and central molecules in *Arabidopsis thaliana* metabolism, serving as precursors for a variety of hormones, but they are not classified as essential in plants [48]. In this functional annotation, the shikimate pathway from erythrose4-phosphate and phosphoenolpyruvate to chorismic acid is a common pathway adopted by *S. cerevisiae* [49] and *A. thaliana* [48] and *Pythium irregulare* (Fig. 4). Tyrosine in *P. irregulare* comes from phenylalanine as found in Diatoms [47] and *M. alpine* [37], instead of 4-Hydroxyphenylpyruvate pathways found in *S. cerevisiae* [49] and *Y. lipolytica* [34] and from L-Arogenate pathway or Phenylalanine in *A. thaliana* [28]. According to Wang et al. [50], this degradation reaction by phenylalanine hydroxylase (EC:1.14.16.1) from phenylalanine to pyrosine is functionally relevant in lipid metabolism, including the sequential reactions to acetyl-CoA (Fig. 4).

##### E – Arginine pathway

According to this functional annotation, glutamate, glutamine, proline, and arginine pathways are derived from 2-oxoglutarate, a product of the TCA cycle. In *P. irregulare,* these pathways can use ammonia, nitrate, or nitrite as a nitrogen source Fig. 4). This provides a great versatility for this microorganism, unlike *S. cerevisiae* and *Y. lipolitica* which cannot consume nitrate and nitrite as a nitrogen sources [34, 35]. Nitrate is present in great amounts in some wastewaters such as vinasse [51].

Besides this difference, the biosynthetic pathways for these amino acids are similar in all studied organisms, except for reaction 16, in pathway E – L-Glutamate, L-Glutamine, Proline, and Arginine described in Fig. 4, which catalyzes the oxidation of Arginine into Citrulline in the presence of NADPH and O_2_ by the family of enzymes named nitric-oxide synthases (NOSs, EC: 1.14.13.39) These are found in *P. irregulare*’s metabolic annotation and *A. thaliana* [28, 52], but not in *S. cerevisiae* [35] and *Y. lipolytica* Arginine is a major storage and transport form of organic nitrogen in plants. Additionally, it has a role in protein synthesis, as a precursor of nitric oxide, polyamines, besides its importance as a pathway in pathogen resistance mechanism [52]. *P. irregulare,* using *A. thaliana as a* host, could develop invasion strategies, including enzyme production to interfere in the metabolic targets common to its host [3], in this case, probably reducing Arginine availability in plants by converting it into Citrulline (by nitric oxide synthase, EC:1.14.13.39) (Fig. 4).

##### Amino acids associated with metabolism of fatty acids (FAs)

Amino acid biosynthesis and degradation play an important role in the biomass development and fatty acids (FAs) biosynthesis, by providing Acetyl-CoA [53], obtained in the degradation of branched-chain amino acid such as leucine (EC:2.3.1.9, EC:4.1.3.4), isoleucine (EC:2.3.1.16), and lysine (EC:1.5.1.8, EC:2.3.1.9) (Fig. 4), as well as in the lysine [54] and L-cysteine biosynthesis [55].

The leucine, isoleucine, and lysine degradation pathways (Fig. 4) are identified in *Y. lipolytica*, an oleaginous microorganism [36], but not in *S. cerevisiae* [35]. Such pathways can be associated to the pathogen’s resistance mechanism, maximizing the energy storage through the accumulation of lipids.

**Fig. 5:**
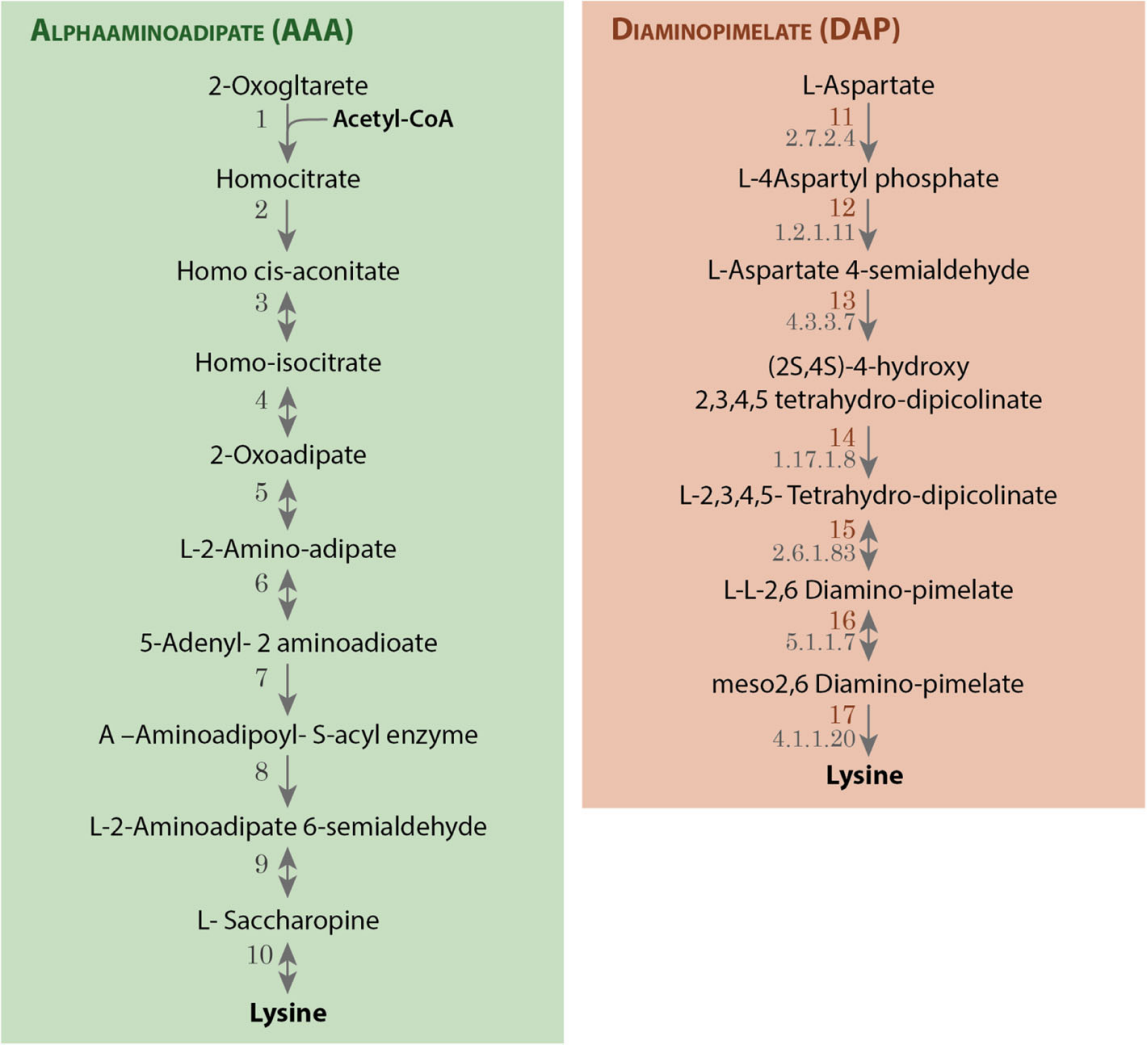
Prediction of Lysine biosynthesis pathways according to the metabolic annotation of *P. irregulare* CBS 494.86. Detailed legend: Diaminopimelate (DAP) possible Lysine biosynthesis pathways with EC numbers provided in the metabolic annotation of *P. irregulare* and Alphaaminoadipate (AAA) pathway available for *S. cerevisiae* [50, 51]. In the DAP pathway enzymes 15 and 16 was not confirmed by the current annotation

Lysine biosynthesis, in *P. irregulare*, comes from the diaminopimelate (DAP) pathway, observed in plants [56] and in other Stramenopiles [47], instead of the alphaaminoadipate (AAA) pathway found in *S. cerevisiae* and *Y. lipolytica*. In DAP, the acetyl-CoA precursor of fatty acids biosynthesis is not consumed [54] (Fig. 5).

**Fig. 6:**
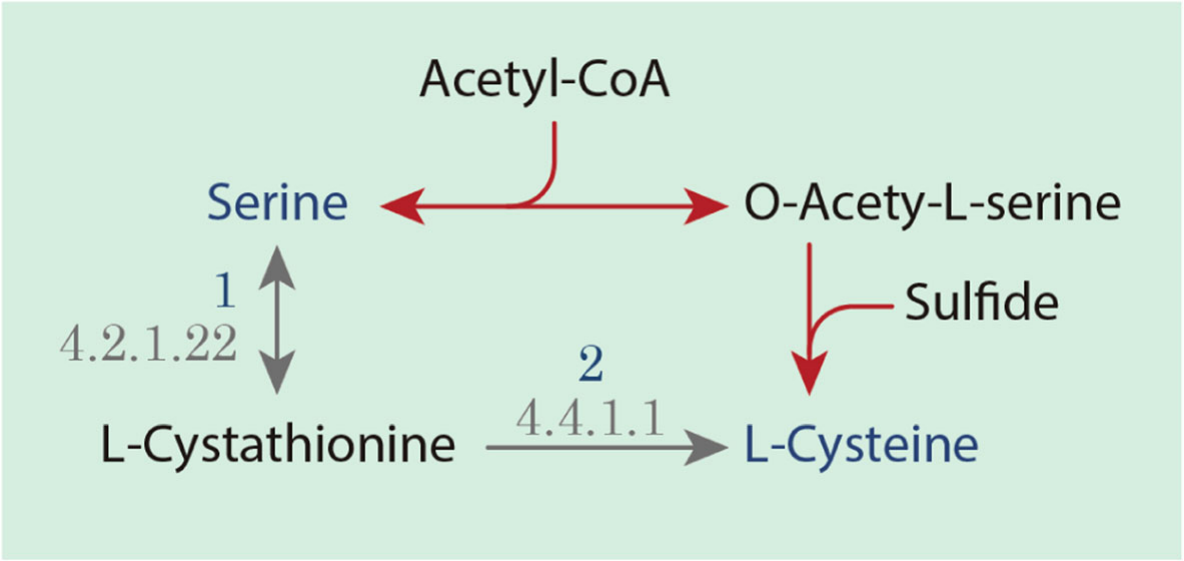
Prediction of Cysteine biosynthesis pathways according to the metabolic annotation of *P. irregulare* CBS 494.86. Detailed legend: The black arrows represent cystathionine (CT) pathway, the most probable pathway applied by *P irregulare* CBS 494.86 according to its metabolic annotation; red arrows represent the O-acetylserine (OAS) pathway

The functional annotation of cysteine biosynthesis is guided to the cystathionine (CT) pathway, also observed in *Phytophthora infestans* which is an oomycete [55], instead of the O-acetylserine (OAS) pathway like in *Arabidopsis thaliana* [28] and other Stramenopiles, such as *T. pseudonana* and *P. tricornutum* [47] (Fig. 6). In the OAS pathway, sulfide is an important metabolite produced in the sulfate assimilation process and its reduction, obtained in plants and diatoms prokaryotes, fungi, and photosynthetic organisms [57]. Acetyl-CoA is used in the production of o-acetylserine (L-Serine + Acetyl-CoA < => O-Acetyl-L-serine + CoA), reducing its avaiability for the biosynthesis of fatty acids (Fig. 6).

#### Fatty acids, unsaturated fatty acids, including EPA

Several Oomycetes, in which *P. irregular* is included, are plant pathogens. They are persistent to several pesticides, due to their ability to store energy in the form of lipids, as their degradation provides acetyl-CoA for further catabolism by the TCA cycle [58]. For this reason, lipids metabolism, including fatty acids biosynthesis, has been thoroughly studied [59]. Moreover, fatty acids are essential compounds in the cell structure of Oomycetes and play an important function in the cell membrane due to the hydrophobic nature of acyl chains, which create subcellular compartments. Moreover, some fatty acids, like polyunsaturated fatty acids, can be used as precursors of eicosanoids that regulate inflammatory and immune responses [60].

FAs are the basic elements of complex lipids (phospholipids, triacylglycerols, sphingolipids, sterol esters) [61]. In the biosynthesis of fatty acids, up to C16 or C18 saturated FAs are produced. This pathway involves two enzymatic systems: type I fatty acid synthase (I FAS), in which enzymes are encoded by a distinct gene, as occurs in most bacteria as well as in the organelles of prokaryotic ancestry, mitochondria and chloroplasts [62]; and type II FAS [63], an enzymatic complex, composed of two subunits, Fas1 (Fasβ) and Fas2 (Fasα) [64], which are found in mammals and lower eukaryotes.

The fatty acid biosynthesis in *P. irregulare* reveals high similarity with *S. cerevisiae, Y. lipolytica, and M. alpina,* which also possess type I and II FAS enzyme systems, instead of *A. thaliana* that possess only I FAS enzymes [28, 31–37].

**Fig. 7:**
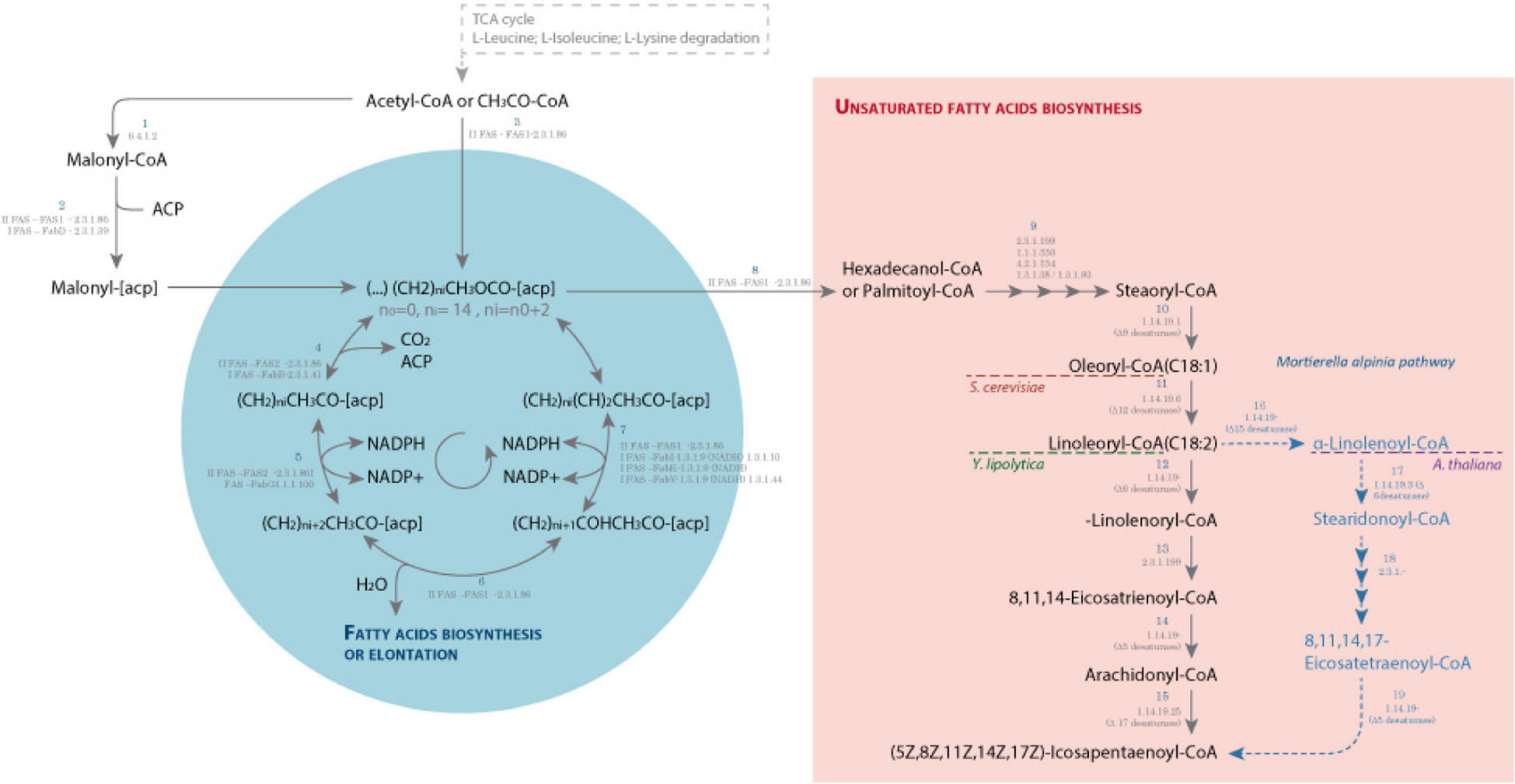
Biosynthesis and unsaturation of fatty acids until EPA predicted from the metabolic annotation. Detailed legend: The biosynthesis predicted from the metabolic annotation in *P. irregulare* CBS 494.86 occurs in the cyclic process by elongation from 2C (Acetyl-acp) to 16C (Hexadecanol-acp), where two carbons from Malonyl-acp are incorporated in the fatty acids per cycle. The process involves fatty acid enzymes from types I (IFAS) and II (II FAS). The enzymatic complex II FAS is composed of two subunits Fas1 (or Fasβ) and Fas2 (or Fasα). The cyclic process involves 4 steps: initiation/elongation (where two carbons of Malonyl-acp are incorporated); reduction (where the first NADPH is reduced to NADP+); dehydration (where there is H2O liberation); and reduction (where the second NADPH is reduced to NADP+). The cyclic process is concluded when Hexadecanol-acp is produced, and converted into Hexadecanol-CoA by the FAS2 enzyme, fatty acid synthase subunit beta (2.3.1.86). The next steps involve unsaturation, by elongation and desaturation processes. The representative figure shows (5Z,8Z,11Z,14Z,17Z)-Icosapentaenoyl-CoA conversion into EPA by long-chain-fatty-acid-CoA ligase and its export by 2.A.126# transporter family. The red dashed line marks the end unsaturated fatty acid (C18:1) produced by *Saccharomyces cerevisiae*; the green dashed line the *Yarrowia lipolytica* end fatty acid (γ-linolenic acid - C18:2); the purple dashed line the *A. thaliana* end fatty acid (α-linoleic acid - C18:3); and the blue dashed lines, metabolites, encoded enzymes, and reactions, which demonstrate the *M. alpine* pathway into EPA

In fatty acid biosynthesis, acetyl-CoA is carboxylated into malonyl-CoA (acetyl-CoA carboxylase; EC:6.4.1.2, Gene: PIR_06001.1), then malonyl is transferred to an acyl-carrier protein (ACP) (fatty acid synthase subunit beta, fungi type, EC:2.3.1.86 or ACP S-malonyltransferase, EC:2.3.1.39). The fatty acid biosynthesis involves 4 reactions, in cyclic steps, which allow producing saturated fatty acids with 16 and 18 carbons (Fig. 7), described next: **initiation/elongation** (3-oxoacyl-[acyl-carrier-protein] synthase I, EC:2.3.1.41, or fatty acid synthase subunit alpha, EC:2.3.1.86), **reduction** (3-oxoacyl-[acyl-carrier protein] reductase, EC:1.1.1.100 or fatty acid synthase subunit alpha, EC:2.3.1.86), **dehydration** (fatty acid synthase subunit beta, fungi type, EC:2.3.1.86), and **reduction** (enoyl-[acyl-carrier protein] reductase I, EC:1.3.1.9 or enoyl-[acyl-carrier protein] reductase II, EC:1.3.1.9 or enoyl-[acyl-carrier protein] reductase / trans-2-enoyl-CoA reductase (NAD+) EC:1.3.1.9 EC:1.3.1.44, or fatty acid synthase subunit beta, fungi type EC:2.3.1.86) (Fig. 7).

The biosynthesis of unsaturated fatty acids involves steps of desaturation and elongation (Fig. 7). In *S. cerevisiae,* the process takes place up to C16:1 and C18:1 by ∆-9 desaturase [58]. Wide-type *Y. lipolytica* can synthetize linoleic acid (C18:2) using a ∆-12 desaturase [34, 65]. *A. thaliana* synthetizes Gamma-linolenic acid (18:3, Omega-6) [66]. *M. alpina* can produce a low amount of EPA (C20:5) (Fig. 7, by its precursor, (5Z,8Z,11Z,14Z,17Z)-Icosapentaenoyl-CoA), but in a possible non-efficient process, which involves sequential conversions from linoleoyl-CoA (Omega 6) to α-linolenoyl-COA (Omega 3) by ∆-15desaturase, from α-linolenoyl-COA to stearidonoyl-CoA by ∆-6 desaturase, Stearidonoyl-CoA into Eicosatetraenoyl-CoA by fatty acid elongase, finally using the Eicosateraenoyl-CoA as a precursor in its production by ∆-5 desaturase [37] (Fig. 7).

Concerning the ability of *P. irregulare* to produce unsaturated fatty acids, the annotation from *P. irregulare* predicted the presence of a gene that encodes the enzyme ∆17 desaturase (EC:1.14.19.-), which converts arachidonyl-CoA (precursor of arachidonic acid) into (5Z,8Z,11Z,14Z,17Z)-icosapentaenoyl-CoA, and finally its conversion into eicosapentaenoic acid) [67–69], which has been reported previously in Oomycetes, such as *Pythium aphanidermatum*, *Phytophthora sojae*, and *Phytophthora ramorum* [67]. Other genes associated with elongation enzymes from palmitoyl-CoA to steaoryl-CoA Fig. 7) and β-oxidation (EC: 1.3.3.6, 4.2.1.17, 1.1.1.211, 2.3.1.16) from eicosapentaenoic acid to docosahexaenoic acid (DHA) were identified. However, there is no evidence of DHA production by *P. irregulare*, according to the review developed by Wu et al. [45].

## Discussion

Table 4 summarizes the divergence in the functional annotation of *P. irregulare* compared with curated information for *A. thaliana*, *S. cerevisiae*, *Y. lipolytica,* and *M. alpina* [28, 31–37]. The metabolic annotation of *P. irregulare* showed high similarity with *A. thaliana*, mainly in carbohydrate metabolism, amino acids metabolism, and nitrogen assimilation, except by the photorespiratory pathways not expected in an Oomycete. Those results could be explained by the horizontal gene and chromosome transfer between *P. irregulare* and its host *A. thaliana* [70]. According to the metabolic annotation, the fatty acid metabolism have high similarity with the fungi analyzed in this article, as producers of lipids with commercial biotechnological interest. However, for the assessed fatty acids, only *M. alpina* is able to produce EPA, but through a different pathway. EPA production with *P. irregulare,* by enzyme Δ-17 fatty acids’ desaturase suggested by the metabolic annotation, provides a great commercial advantage, as this desaturase can use fatty acids both from the acyl-CoA fraction and the phospholipids fraction as substrates [67].

**Table 4:** Key differences observed in *Pythium irregulare* metabolic annotation compared with *other microorganisms*^*a*^

Pathways	*Pyhtium irregulare* metabolic annotation (this study)	*Arabidopsis thaliana,*	*Saccharomyces cerevisiae*	*Yarrowia lipolytica*	*Mortierella alpina*
*Carbohydrate metabolism*					
*Starch and Sucrose metabolism*					
Cellulose consumption	√	√	–	–	–
*Fatty acids metabolism*					
*Fatty acids biosynthesis*					
Fatty acids synthase type I (IFAS)	√	√	√	√	√
Fatty acids synthase type II (IIFAS)	√	–	√	√	√
*Unsaturated fatty acids*					
EPA production	√ EC:1.14.19.-∆17desaturase	–	–	–	√ EC: 1.14.19.-∆5desaturase
*Amino acids*					
*Lysine biosynthesis*					
Diaminopimelate (DAP) pathway	√	√	–	–	–
Alphaaminoadipate (AAA) pathway	–	–	√	√	–
*Serine biosynthesis*					
Non-photorespiratory pathway	√	√	√	–	–
Photorespiratory pathway	–	√	–	–	–
*Cysteine biosynthesis*					
Cystathionine (CT) pathway	√	√	√	√	–
O-acetylserine (OAS) pathway	–	√			
*Arginine biosynthesis*					
Arginine reoxidation into Citrulline	√	√	–	–	–
*Tyrosine biosynthesis*					
Phenylalanine degradation	√	√	–	–	√
*Nitrogen Metabolism*					
Nitrate reduction	√	√	–	–	√
Nitrite reduction	√ EC:1.7.1.4	√ EC:1.7.7.1	–	–	√ EC:1.7.1.4

^a^Curated information

Finally this metabolic annotation can infer about *P. irregulare* metabolic capabilities, supplying information of great importance to future work.

## Conclusions

This unprecedented functional annotation demonstrates the presence of relevant genes and is consistent with results described in literature. Genes associated with the amino acids production, consumption of carbon (glucose, sucrose, cellulose, fructose, glycerol, and others) and nitrogen sources (nitrate, nitrite, and ammonia), present in the wastewater (produced in large amounts around the world) provide great advantage in the production of value-added lipids using low cost carbon source and in an efficient way, for food and pharmaceutical industries. Several genes encode enzyme present in pathways able to maximize lipid production, notably, the enzyme Δ-17 fatty acid desaturase which can use not only fatty acids in acyl-CoA, but also fatty acids in the phospholipid fraction as substrates, providing a competitive advantage for EPA production [67].

This original functional annotation of *Pythium irregulare* can serve as the basis for the reconstruction of a genome scale metabolic model, which can be used to optimize biomass growth and EPA production.

Finally the metabolic annotation process developed in this article can be generalized to any strains and applied as an useful and straightforward tool in the metabolic engineering field.

## Methods

### Microorganism cultivation

*P. irregulare* strain CBS 494.86 was acquired from the CBS-KNAW Fungal Biodiversity Centre. It was inoculated on PDA plates (Potato Dextrose Agar) and incubated for 3 days at 29 °C, then 1 cm^2^ of the grown microorganism was transferred and cultivated on YPDO medium (g/L: yeast extract 1.25, peptone 25, glucose 3, oatmeal 2) for 5 days at 29 °C in a shaker at 200 rpm.

Even though several articles have been published about *P. irregulare* DAOM BR 486 / CBS 250.28 [14, 18, 23, 71], mainly associated with its pathogenicity, with its genome sequences deposited at NCBI (Bioproject number: PRJNA169053), strain *Pythium irregulare* CBS 493.84 had not been explored yet.

### Species validation and genome sequencing

The DNA of the biomass was extracted following the de Graaff et al. protocol [72]. As the first step, to validate the species of this work, two conserved regions were chosen to be analyzed, as proposed by [71]. Cytochrome oxidase I (COI) and the Internal Transcribed Spacer Regions (ITS1 and ITS2) were amplified by PCR using the following set of primers: OomCoxI-Levup (5′-TCAWCWMGATGGCTTTTTTCAAC-3′) and Fm85mod (5′-RRHWACKTGACTDATRATACCAAA-3′), UN-up18S42 (5′-CGTAACAAGGTTTCCGTAGGTGAAC-3′) and UN-lo28S22 (5′-GTTTCTTTTCCTCCGCTTATTGATATG-3′), respectively [73], and submitted for sequencing by Sanger method (Applied Biosystems), following the provider’s protocol. Validating the working species, the genomic DNA libraries for Next Generation Sequencing were produced using the Nextera DNA library preparation kit (Illumina). Sequencing was carried out on a Illumina HiSeq 2500 instrument, using paired-end chemistry (58.990.406 sequenced fragments 2*100 bp) at the NGS core facility of the Brazilian Bioethanol Science and Technology Laboratory (CTBE). K-mer statistics revealed an expected genome size of approx. 75 Mbp [74]. Genome assembly was carried out SPAdes using an ensemble of different k-mer values [75] and improved with Pilon [76]. Ploidy level analysis was carried out with ploidyNGS [77], which revealed that this organism is diploid, thus the inferred genome sequence was processed with Redundans to eliminate redundancies due to allelic polymorphisms. The genome of *P. irregulare* strain (CBS 494.86) is described at the GenBank sequence database provided by the National Center for Biotechnology Information (NCBI) in PRJNA371716.

### Gene prediction and annotation

#### Functional annotation

The phenotypic potential of an organism is embedded in its genome sequence, and gene product identification is compulsory in order to understand the occurring biological processes [78].

The annotation of a genome is the process of identifying and cataloging functional information of genes in a sequenced genome [79]. The important information retrieved from a genome annotation is gene name, assigned cellular functions, and Enzyme Commission (EC) number, for enzyme coding genes [80].

The *merlin*, a user-friendly software tool, was created to assist in the processes of annotation and reconstruction of genome-scale metabolic models, by performing automatic genome-wide functional annotations and providing a numeric score for each automatic assignment, taking into account the frequency and taxonomy within the annotation of all similar sequences [81].

**Fig. 8:**
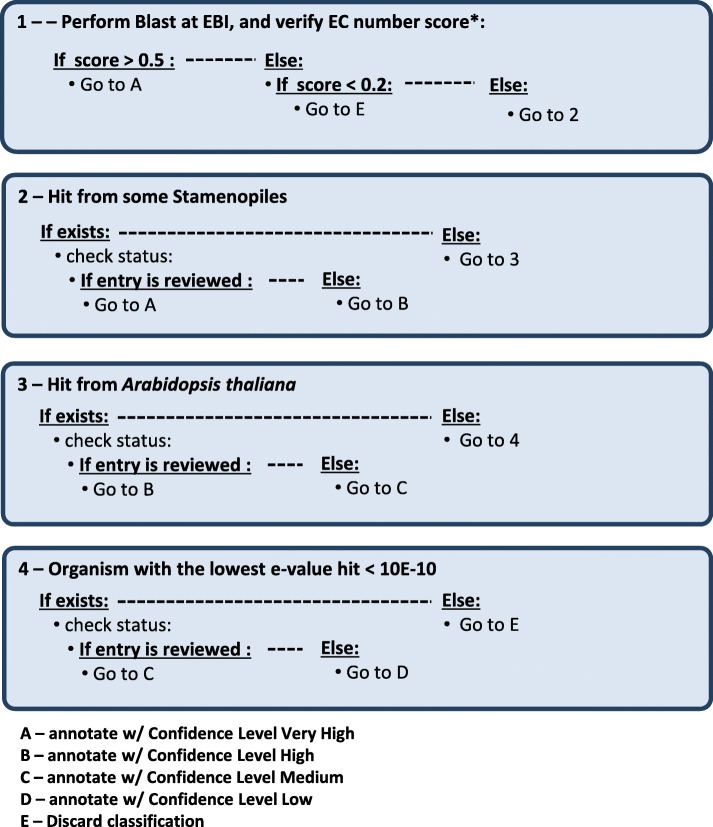
Re-annotation pipeline for manual inspection of each candidate gene in *merlin.* Detailed legend: * The score was calculated using the first 100 priority reviewed homologues retrieved from the BLAST similarity, and the thresholds were manually curated by the authors, described in the methods section

The selection of the best threshold for automatic annotation *in merlin* involved adjusting the alpha-value (a ratio of taxonomy and frequency score) using a set of random manually curated sequences and comparing these with the automatic annotation provided by the software (automatic classification and final metabolic annotation available in Additional files 3 and 4). In this process, the alpha-value in *merlin* was set at 0.9, which emphasises the frequency score, due to the lack of reviewed information on the Stramenopiles lineage. The selected score threshold for automatically accepting annotation in *merlin* was set to 0.5, meaning that any candidate genes with a score higher than 0.5 are automatically annotated. Candidate genes with a score below 0.2 were automatically discarded (Additional file 3). After analysing *merlin*‘s similarity search output, an annotation workflow was developed to classify and curate the annotation. This workflow was developed to systematically analyze *merlin*‘s classification and accept or reject it. The annotation workflow follows a series of simple steps to determine each gene’s classification, together with the confidence level of such annotation. The confidence level was set by the authors. It starts by addressing specific situations with a high confidence level (A) (Fig. 8), extending then the search, covering a larger amount of possible annotations, whilst decreasing the confidence level. The EC number classification and the taxonomic distance of the results are taken into account, as well as reviewed information and literature on the studied gene.

A complete metabolic annotation involves identifying genes encoding enzymes and membrane transporters (Additional file 4).

The transporter candidate genes (TCGs) annotation of *Pythium irregulare* was performed in *merlin*‘s TRIAGE (Transport Proteins Annotation and Reactions Generation) [24].

Initially, protein-encoding genes with transmembrane helices were identified using Phobius [82, 83]. Afterwards, *merlin* runs the Smith-Waterman (SW) algorithm [84] to compare the target TCGs’ translated gene sequences with transmembrane helices with all protein sequences available in the TCDB database. Finally, the metabolites transported by each carrier are inferred from the annotations of the TCDB records that have similarities with thcarrier TCGs [24]. The assessment of the subcellular localization of the proteins was predicted usingLocTree3 [85]. The pipeline of annotating transporter candidate genes (TCGs) of *Pythium irregulare* and the genes associated are available in the Availability of data and materials (Additional files 5 and 6).

## Additional files

Additional file 1: **Figure S1.** Wastewater composition and forecast for 2024 – Vinasse and Glycerol compostion and worldwild forecast production for 2024. (PDF 806 kb)

Additional file 2: Comparison *Pythium irregulare* – Genome comparison between *Pythium irregulare* strains. (XLSX 13 kb)

Additional file 3: Pipeline classification - Pipeline classification for annotation and reconstruction of genome-scale metabolic models established according dataset analysis. (XLSX 1790 kb)

Additional file 4: The metabolic annotation – The metabolic annotation of *Pythium irregulare* CBS 494.86 genome. (XLSX 4245 kb)

Additional file 5: The transporter annotation - Pipeline classification for transporter annotation established according dataset analysis. (XLSX 45 kb)

Additional file 6: Transporter candidate genes - The transporter annotation of *Pythium irregulare* CBS 494.86 genome. (XLSX 63 kb)

## Data Availability

The datasets generated and/or analysed during the current study are available in the PRJNA371716 repository, https://www.ncbi.nlm.nih.gov/bioproject/?term=PRJNA371716. All data generated or analysed during this study are included in this published article and its supplementary Additional files:

## Abbreviations

AAA: Alphaaminoadipate
ACP: Acyl-carrier protein
CAGR: Compound annual growth rate
COI: Cytochrome oxidase I gene
DAP: Diaminopimelate
DHA: Docosahexaenoic acid
EC: Enzyme code
EPA: Eicosapentaenoic acid
FAs: Fatty acids
ITS: Internal transcribed spacer
NCBI: National Center for Biotechnology Information
OAS: O-acetylserine
TC: Transporter classification
TCA: Tricarboxylic acid
