# Supplementary material for: Genome-wide sequencing and metabolic annotation of Pythium irregulare CBS 494.86: understanding Eicosapentaenoic acid production
Source: BMC Biotechnol. 2019 Jun 28;19:41. doi: 10.1186/s12896-019-0529-3 (PMC6598237; doi:10.1186/s12896-019-0529-3)
Supplement: Supplementary file 1 — Figure S1. Wastewater composition and forecast for 2024 – Vinasse and Glycerol compostion and worldwild forecast production for 2024. (PDF 806 kb) [file 12896_2019_529_MOESM1_ESM.pdf]

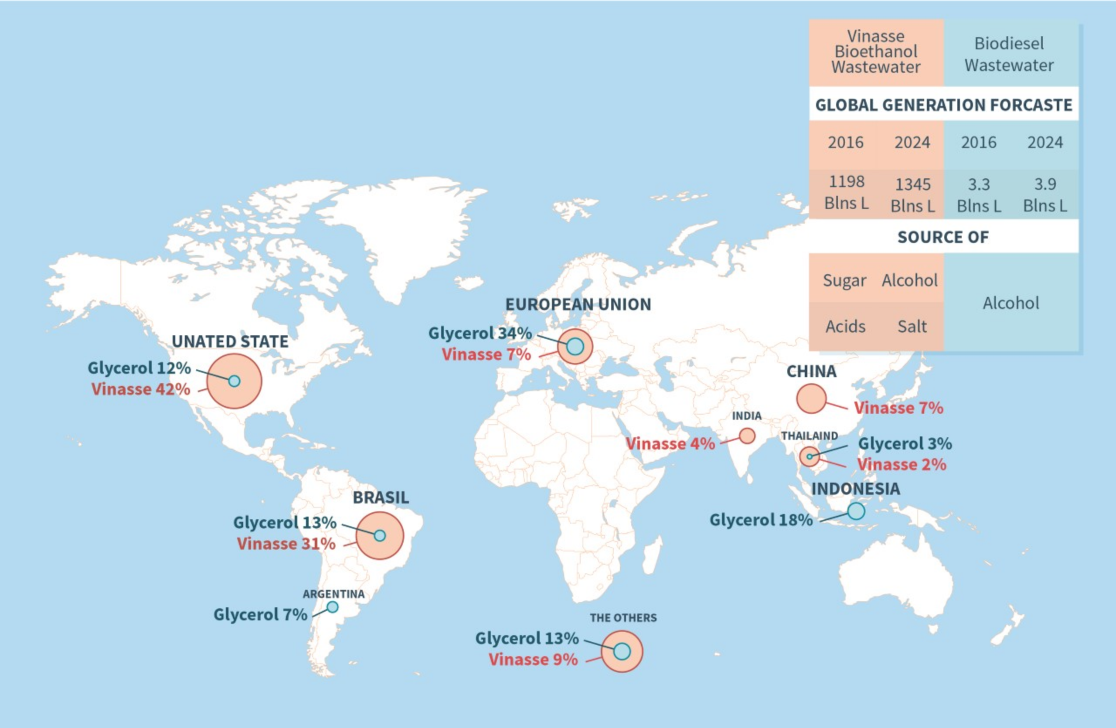

Figure 1. Vinasse, bioethanol wastewater, and biodiesel wastewater globally generation forecast for 2024 according to OECD/FAO (1,2) and their composition. (Estimative of volumetric production per year – vinasse production = 10 x bioethanol production, biodiesel wastewater = biodiesel / 10; Vinasse composition - Sugar: sucrose, glucose, fructose, arabinose, cellulose; salts: phosphate, nitrate, ammonium; Acids: acetate, citrate, oxalate, formiate; Alcohol: glycerol, ethanol. Biodiesel wastewater composition – Alcohol: glycerol) Source: owned by authors

1. OECD/FAO. Biofuels. Agric outlook [Internet]. 2015;144. Available from: http://www.oecd-ilibrary.org/agriculture-and-food/oecd-fao-agricultural-outlook-2015/biofuels_agr_outlook-2015-13-en

2. FAO. OECD-FAO Agricultural outlook 2016-2025 [Internet]. 2016. Available from: http://www.oecd-ilibrary.org/agriculture-and-food/oecd-fao-agricultural-outlook-2016_agr_outlook-2016-en
